# Editorial: The Triple Interaction: Diet, Microbiota and Epigenetics in the Onset and Management of Type 1 Diabetes

**DOI:** 10.3389/fnut.2021.705770

**Published:** 2021-09-17

**Authors:** Annalisa Terranegra, Goran Petrovski, Elvira Verduci

**Affiliations:** ^1^Research Department, Sidra Medicine, Doha, Qatar; ^2^Endocrinology Clinic, Sidra Medicine, Doha, Qatar; ^3^Department of Pediatrics, Vittore Buzzi Children's Hospital, University of Milan, Milan, Italy; ^4^Department of Health Sciences, University of Milan, Milan, Italy

**Keywords:** diet, microbiota, epigenetics, type 1 diabetes, therapy

## Introduction

Type 1 diabetes (T1D) is an autoimmune disease that leads to the destruction of pancreas ß-cells and insulin deficiency. The main determinant of T1D is genetic predisposition, although most children born carrying T1D genetic risk markers do not develop the disease and, as discussed in most of the papers in this collection, genetics cannot fully explain the disease pathogenesis (Hamilton-Williams et al.; Verduci et al.; Kohil et al.; Al Theyab et al.).

It becomes more and more apparent that T1D is a complex disease with intermediate phenotypes, many comorbidities, and heterogeneity in its therapeutic responses. This complexity can be due to interaction with diet and lifestyle that ultimately can affect the microbiota and host immune system *via* epigenetic mechanisms.

The Research Topic represented a fantastic opportunity for the scientific community to provide up-to-date knowledge and propose mechanisms explaining the role of gut microbiota as an epigenetics effector in T1D, and how diet and lifestyle can modulate this interaction, opening new opportunities in the diagnosis and management of T1D. Three key areas have been investigated:

1. Role of diet in T1D pathogenesis
2. Epigenetic function of microbiota
3. Therapeutic prospective.

## Diet and T1D Pathogenesis

Different types of diets may have a protective role against T1D, among those the gluten-free diet (Al Theyab et al.; Hamilton-Williams et al.) and the Mediterranean diet (MD), thanks to its anti-inflammatory properties (Calabrese et al.). On the contrary, a diet high in sugar can increase the risk of T1D, replacing fibers with sugar, which has a detrimental effect on the gut microbiota (Hamilton-Williams et al.). Confirmatory data comes from trials with high fiber diets that showed improvement in the BMI and blood pressure of T1D patients (Kohil et al.). Diets enriched in short-chain fatty acids (SCFA), (Al Theyab et al.) as well as in omega-3 fatty acids (Hamilton-Williams et al.; Kohil et al.), have been reported as potentially beneficial for T1D and islet autoimmunity (IA) by improving bacterial composition. Among the micronutrients, vitamin A deficiency alters the ratio of Firmicutes to Bacteroides and increases the inflammatory response. Iron and zinc also affect microbial composition and the inflammatory response (Al Theyab et al.). Surprisingly, there are contrasting findings on the effect of vitamin D deficiency in T1D (Kohil et al.) and on the gut microbiota (Hamilton-Williams et al.; Calabrese et al.). All these mechanisms can link diet with the etiopathogenesis of T1D, with special attention to early nutrition and specific dietary patterns, such as MD and gluten-free diets, can be explored as therapeutic approaches.

### Early Nutrition

T1D development has been shown to be affected by maternal diet during pregnancy, breastfeeding, and the early introduction of certain foods in infancy. Gut microbiota is also strongly influenced by these factors and their modification impacts on the immune system. A review of the current literature by Verduci et al. encourages long-term breastfeeding and a careful introduction of complementary foods (at 4 months) and cows' milk (at 12 months) to reduce the risk of T1D. Potential mechanisms include the breastmilk nutrients and hormones [human milk oligosaccharides (HMO), leptin, insulin, etc.] that contribute to the maturation of the immune system and the gut microbiota, the increased gut permeability and gut inflammation associated with the early introduction of cow milk vs. the intake of fermented milk products that increase the genera *Bifidobacteria* and improve the glycemic indexes in animal diabetes models, and finally complementary foods which also accelerate microbiota maturation and diversity impacting on the gut barrier permeability (Verduci et al.). The early gluten introduction also takes a central place in the discussion on the role of diet in T1D pathogenesis, with many common mechanisms between T1D and celiac disease (Verduci et al.; Hamilton-Williams et al.).

### Gluten-Free Diet

The gluten-free diet seems to attenuate the autoimmune response by increasing the number of beneficial bacteria *via* mechanisms common with coeliac disease. Hamilton-Williams et al. summarized the contrasting findings from different studies, such as the Diabetes Autoimmunity Study in the Young (DAISY) that did not find any association of gluten intake with IA and T1D, and two other studies in Finland and Norway that found an association of gluten intake with IA and T1D, respectively. Both Hamilton-Williams et al. and Al Theyab et al. reported on clinical trials suggesting a protective role of the gluten-free diet in animal models. Non-obese diabetic (NOD) mice fed with a gluten-free diet show a decreased risk of developing T1D, an increase in T regulatory cells, and a modification of the gut microbiota (reduction of *Bifidobacterium, Tannerella*, and *Barnesiella* species, and increase of *Akkermansia*).

### Mediterranean Diet

The MD, known for its benefits on health, is explored as a potential therapeutic strategy to prevent and delay T1D progression, also *via* modulating the gut microbiota. The MD ensures a balanced intake of fruits, vegetables, whole-grain cereals, legumes, and unsaturated fats. These foods are able to improve the microbiota and gut health, increasing the level of SCFA, particularly butyrate, and reducing the levels of TMAO, in addition to the high intake of fibers that improve glycemic control and absorption of fats. These mechanisms are discussed by Calabrese et al., suggesting the beneficial effect of the MD approach to T1D and its cardiovascular complications.

## The Epigenetic Function of Microbiota

The role of epigenetic mechanisms in the pathogenesis and progression of T1D is a new concept that is attracting wide interest. Diet and gut microbiota are linked to epigenetics in a complex interaction. Kohil et al. reviewed all the epigenetic mechanisms potentially involved in the pathogenesis of T1D, such as the hypermethylation of Insulin (INS) and Interleukin 2 receptor (IL2R) genes identified in T1D patients, and other methylated DNA regions associated with both diabetic nephropathy and pancreas development. Some of the histone modifications and microRNAs are also identified as associated with T1D and its complications. High fat and high carb diets, as well as specific nutrients (folate, vitamin B12, LC-PUFA, flavonoids, etc.) are involved in the epigenetic modifications that can lead to T1D or other metabolic disorders. Al Theyab et al. proposed a complex pathway that involves microbial metabolites, particularly SCFA, in the epigenetic modifications leading to T1D. Among the SCFA, the butyrate exerts a protective effect by inhibiting the histone deacetylases (HDAC), blocking the inflammatory pathways, and improving the pancreatic tissue damage; the acetate as well has been tested in NOD mice and resulted in a reduced diabetic rate and insulitis increasing the expression of genes involved in the differentiation of the Treg cells. Polyphenols and vitamins, that are also metabolized by the gut microbiota from the ingested food, are known to be epigenetic modulators and cofactors.

From a different perspective, Giampaoli et al. discussed in a mini review the potential interaction between the genetic background of the fucosyltransferase 2 (FUT2) gene and gut microbiota in T1D. FUT2 is involved in the expression of H antigen precursor of ABH histo-blood group antigen expressed in bodily fluids including the intestinal mucosa. Its genetic variants determine the loss of expression of ABH antigens in the gastrointestinal tract, called “*non-secretors*” and correlated with T1D risk. The *non-secretor* status is associated with reduced microbial diversity, depleted microbial amino-acid metabolism, as well as enriched carbohydrate and lipid metabolism, and sub-clinical intestinal inflammation. The maternal FUT2 gene may also play a role in infant gut colonization, modifying the content of HMO, particularly in fucosylated oligosaccharides. Milk from *non-secretor* mothers lacks in fucosylated oligosaccharides, and infants breastfed by *non-secretor* mothers show a lower content of Bifidobacteria, similar to T1D pediatric patients. The authors suggest that a FUT2-dependent mechanism may likewise take place in T1D patients due to a complex interaction among genetic background, maternal factors, and microbiota.

## The Therapeutic Prospective

Many authors in this Research Topic highlighted the potential of modulating the gut microbiota as a therapeutic approach to T1D. Al Theyab et al. summarized the studies of probiotics (mainly *Lactobacillus* spp), prebiotics (inulin, resistant starch, caffeic acid, hydrolyzed casein, human milk oligosaccharides, etc.), and symbiotics tested mainly in diabetes animal models. Hamilton-Williams et al. presented the ongoing clinical trials testing pre- and pro-biotics in T1D patients. In addition, Hamilton-Williams et al. also discuss the use of fecal microbiota transplantation (FMT) in adult T1D patients and the development of the “next-generation probiotics” indigenous to the human gut that are expected to be more efficient in the treatment of T1D. MD, as already mentioned, was discussed by Calabrese et al. for its potential application as diet therapy for T1D. Verduci et al. elaborated on the potential of diet, probiotic, and prebiotic treatment in pediatrics T1D patients. The Authors mainly reported on studies on probiotics tested in the 1st days and months of life, with contrasting results, with fewer studies on prebiotics and postbiotics in animal models. Moreover, they presented interventional studies using a gluten-free diet, vitamins, and minerals in pediatric patients and animal models which modified the microbial composition and improve diabetes indices in the same cases. Last but not least, of note is the suggestion of supplementing T1D pediatric patients carrying FUT2 genetic variants with a-(1, 2)-fucosyl oligosaccharides in formula milk (Giampaoli et al.).

Overall, new perspectives are open on the role of diet in affecting gut microbiota and its function as an epigenetic effector and how these factors can be targeted for new therapies in T1D.

## Author Contributions

AT drafted the Editorial. EV and GP revised it. All authors approved it for publication.

## Conflict of Interest

The authors declare that the research was conducted in the absence of any commercial or financial relationships that could be construed as a potential conflict of interest.

## Publisher's Note

All claims expressed in this article are solely those of the authors and do not necessarily represent those of their affiliated organizations, or those of the publisher, the editors and the reviewers. Any product that may be evaluated in this article, or claim that may be made by its manufacturer, is not guaranteed or endorsed by the publisher.

